# Effects of multimodal exercise program on postural balance in patients with chronic obstructive pulmonary disease: study protocol for a randomized controlled trial

**DOI:** 10.1186/s13063-023-07558-9

**Published:** 2023-08-15

**Authors:** Tamires Daros dos Santos, Adriane Schmidt Pasqualoto, Dannuey Machado Cardoso, Ivana Beatrice Mânica Da Cruz, Rafael Noal Moresco, Aron Ferreira da Silveira, Isabella Martins de Albuquerque

**Affiliations:** 1 Programa de Pós-Graduação em Distúrbios da Comunicação Humana, Universidade Federal de Santa Maria (UFSM), Avenida Roraima, 1000, Santa Maria, 97105-900 Brazil; 2 Universidade Federal do Rio Grande do Sul (UFRGS), Porto Alegre, Rio Grande do Sul 90010-150 Brazil; 3 Centro de Ensino Superior Dom Alberto, Santa Cruz do Sul, Brazil; 4 Programa de Pós-Graduação em Farmacologia e Programa de Pós-Graduação em Gerontologia, Universidade Federal de Santa Maria (UFSM), Avenida Roraima, 1000, Santa Maria, 97105-900 Brazil; 5 Programa de Pós-Graduação em Ciências Farmacêuticas, Universidade Federal de Santa Maria (UFSM), Avenida Roraima, 1000, Santa Maria, 97105-900 Brazil; 6 Programa de Pós-Graduação em Ciências do Movimento e Reabilitação, Universidade Federal de Santa Maria (UFSM), Avenida Roraima, 1000, Santa Maria, 97105-9000 Brazil

**Keywords:** Breathing exercises, Electric stimulation, Postural balance, Rehabilitation, Randomized controlled trial

## Abstract

**Background:**

Evidence has shown that patients with chronic obstructive pulmonary disease present significant deficits in the control of postural balance when compared to healthy subjects. In view of this, it is pertinent to investigate the effects of different therapeutic strategies used alone or in association with pulmonary rehabilitation with the potential to improve postural balance and other outcomes with clinical significance in patients with chronic obstructive pulmonary disease. This study will investigate the effects of an 8-week (short-term) multimodal exercise program [inspiratory muscle training (IMT) plus neuromuscular electrical stimulation (NMES)] on postural balance in patients with chronic obstructive pulmonary disease enrolled in a pulmonary rehabilitation program compared to individualized addition of IMT or NMES to pulmonary rehabilitation or standard pulmonary rehabilitation.

**Methods:**

This is a randomized, single-blind, 4-parallel-group trial. Forty patients with chronic obstructive pulmonary disease will be included prospectively to this study during a pulmonary rehabilitation program. Patients will be randomly assigned to one of four groups: multimodal exercise program (IMT + NMES + pulmonary rehabilitation group) or (IMT + pulmonary rehabilitation group) or (NMES + pulmonary rehabilitation group) or standard pulmonary rehabilitation group. Patients will receive two sessions per week for 8 weeks. The primary outcome will be static postural balance and secondary outcomes will include as follows: static and dynamic postural balance, fear of falling, muscle strength and endurance (peripheral and respiratory), functional capacity, health-related quality of life, muscle architecture (quadriceps femoris and diaphragm), and laboratory biomarkers.

**Discussion:**

This randomized clinical trial will investigate the effects of adding of short-term multimodal exercise program, in addition to pulmonary rehabilitation program, in postural balance in patients with chronic obstructive pulmonary disease enrolled in a pulmonary rehabilitation. Furthermore, this randomized control trial will enable important directions regarding the effectiveness of short-term intervention as part of the need to expand the focus of pulmonary rehabilitation to include balance management in chronic obstructive pulmonary disease patients which will be generated.

**Trial registration:**

ClinicalTrials.gov NCT04387318. Registered on May 13, 2020.

## Background and rationale {6a}

### Background

Oxidative stress is involved in the pathogenesis and progression of chronic obstructive pulmonary disease (COPD) causing cell damage [1]. In this scenario, clinical studies demonstrate in DNA a significant increased damage and inefficiency in repair mechanisms which may lead to genomic instability and cellular senescence in patients with COPD [2]. The primary pathophysiology of COPD is related to damage to the respiratory system [3]. However, the respiratory and limb muscle dysfunction appears to be a major systemic manifestation in COPD [4], contributing to reduction in functional capacity and health-related quality of life (HRQoL) [5].

A recent meta-analysis showed that compared with healthy controls, patients with COPD have a clinically meaningful balance reduction [6]. Balance impairment is one of the main risk factors for falls, and patients with COPD are four times more likely to fall than their healthy peers, leading to increased morbidity and mortality [6, 7].

Because COPD is a complex and systemic disease, its treatment requires an integrated therapeutic approach, including the referral for pulmonary rehabilitation (PR) [3]. PR has been demonstrated to reduce dyspnea and improve exercise capacity and HRQoL; its core components include aerobic and strength training [8, 9]. However, despite accumulating evidence on balance impairment in patients with COPD, routine assessment of this outcome is still not an included in PR [8, 9]. The American Thoracic Society (ATS)/European Respiratory Society (ERS) Statement on PR briefly presents a broadened scope of outcomes assessments in PR, including balance. Nevertheless, the focus of PR should be expanded to include the assessment, training, and elucidation of the potential mechanisms underlying balance deficits in COPD [6].

Respiratory and limb muscle dysfunctions are particularly relevant in the COPD because they contribute to balance impairments [4, 6, 10]. In this sense, it is worth noting that the diaphragm plays an important role in postural balance and the impairment function of this muscle observed in COPD contributes to the deterioration in balance [11]. Therefore, it is reasonable to infer that a multimodal exercise program [inspiratory muscle training (IMT) plus neuromuscular electrical stimulation (NMES)] could potentially improve postural balance. These therapeutic strategies have been used to complement PR [12, 13]. However, no study has assessed the efficacy of short-term multimodal exercise programs in addition to PR in patients with COPD.

## Objectives {7}

Therefore, this study aims to investigate the effects of short-term multimodal exercise program (IMT plus NMES) compared to individualized addition of IMT or NMES to PR or standard PR on postural balance, fear of falling, muscle strength and endurance (peripheral and respiratory), functional capacity, HRQoL, muscle architecture (quadriceps and diaphragm), inflammatory profile, endothelial function, oxidative stress and DNA damage in patients with COPD. We hypothesize that the addition of a multimodal exercise program could potentiate the aforementioned outcomes.

## Trial design {8}

This is a protocol for a clinical, randomized, controlled, single-blind (outcomes assessor), four-arm parallel-group, and framework superiority study. Patients will be randomized (1:1:1:1). This study was approved by the ethics committee (process no. 3.208.982). The protocol will be conducted according to the Consolidated Standards of Reporting Trials (CONSORT) statement. The trial was registered at ClinicalTrials.gov (“X”).

## Methods: participants, interventions, and outcomes

### Study setting {9}

This will be a single-center study carried out at the PR program at the University Hospital of Santa Maria, Rio Grande do Sul, Brazil.

### Eligibility criteria {10}

The eligibility criteria include patients aged 55 years or more (adult, older adult) of both sexes.

#### Inclusion criteria

1. Clinical diagnosis of COPD, stages II, III, or IV [3];
2. Clinically stable, i.e., absence of infections or exacerbations in the last 3 months;
3. Medical clearance to participate in PR;
4. Availability of attending to the PR.

#### Exclusion criteria

1. Unstable primary pathologies (cardiovascular, renal, metabolic, or psychiatric);
2. Nutritional aspects (nutritional supplementation on the 4 weeks preceding the study or obesity (BMI>30 kg/m^2^));
3. Severe hearing, visual or vestibular disorder impairment recorded on patient chart or self-referred;
4. Evidence of a neurological or musculoskeletal condition that severely limits mobility and postural control;
5. Contraindications to NMES;
6. Participation in PR programs in the 3 months previous to the study or physically active;
7. Active smoker and/or active alcohol;
8. Cognitive impairment.

**Fig. 1:**
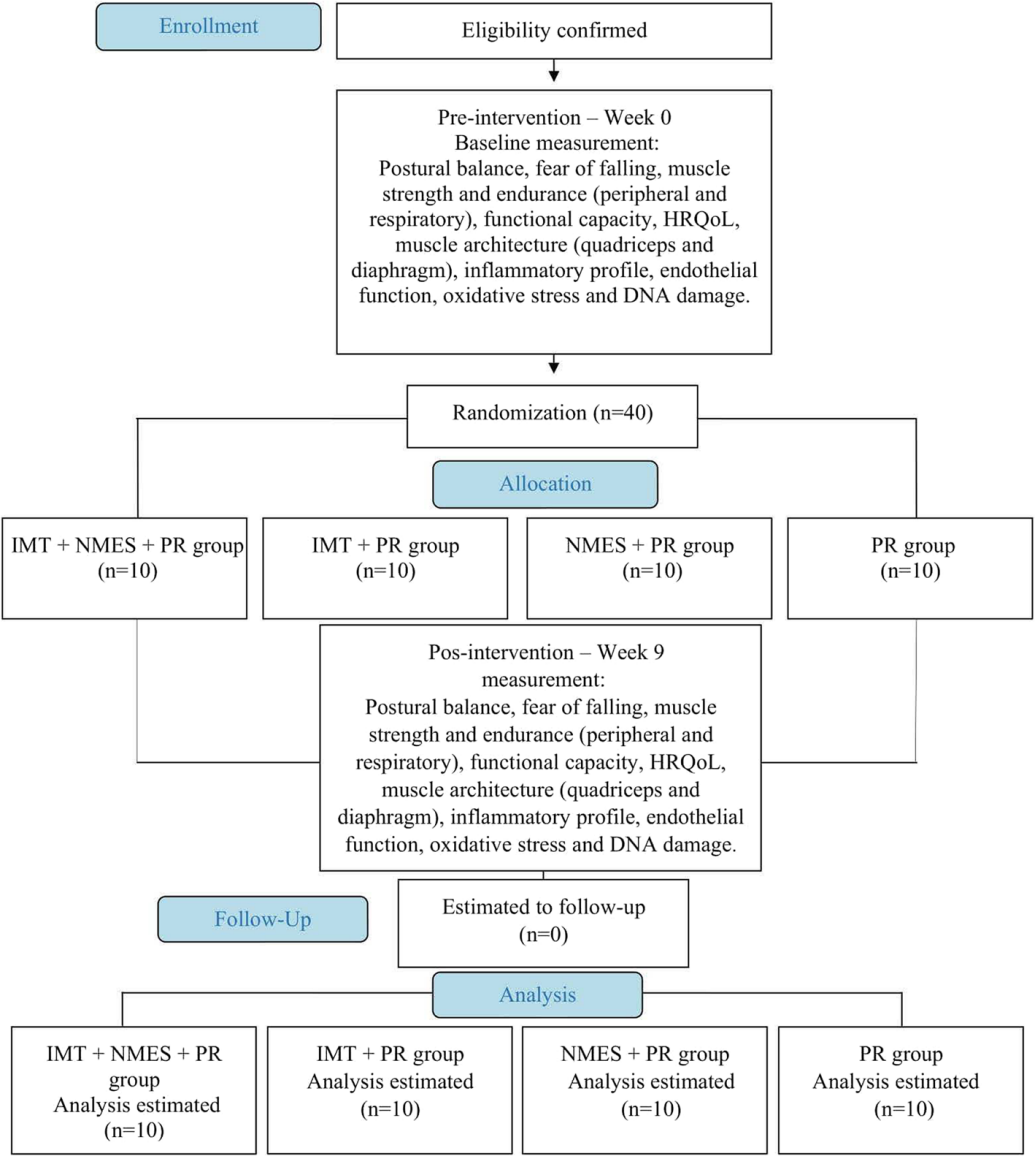
CONSORT flowchart of the planned protocol pathway. HRQoL: Health-related quality of life; IMT: Inspiratory muscle training; NMES: Neuromuscular electrical stimulation; PR: Pulmonary rehabilitation

All eligible patients will undergo the same assessments at the beginning and after 8 weeks of the study (Fig. 1).

### Who will take informed consent? {26a}

The TDS researcher will be in charge of contacting potential participants whose names are on the waiting list to start PR and invite them to participate in the study. If we have a positive reply, an appointment will be scheduled, and the TDS will explain the objectives of the study, risks, benefits, and ethical implications. If participants agree to participate in the study, they will be asked to sign two copies of an informed consent form: one for the participants and the other for the researcher.

### Additional consent provisions for collection and use of participant data and biological specimens {26b}

We will request consent from participants for the collection of blood samples to assess biomarkers, endothelial function, oxidative stress, muscular damage, and DNA damage.

## Interventions

### Explanation for the choice of comparators {6b}

**Fig. 2:**
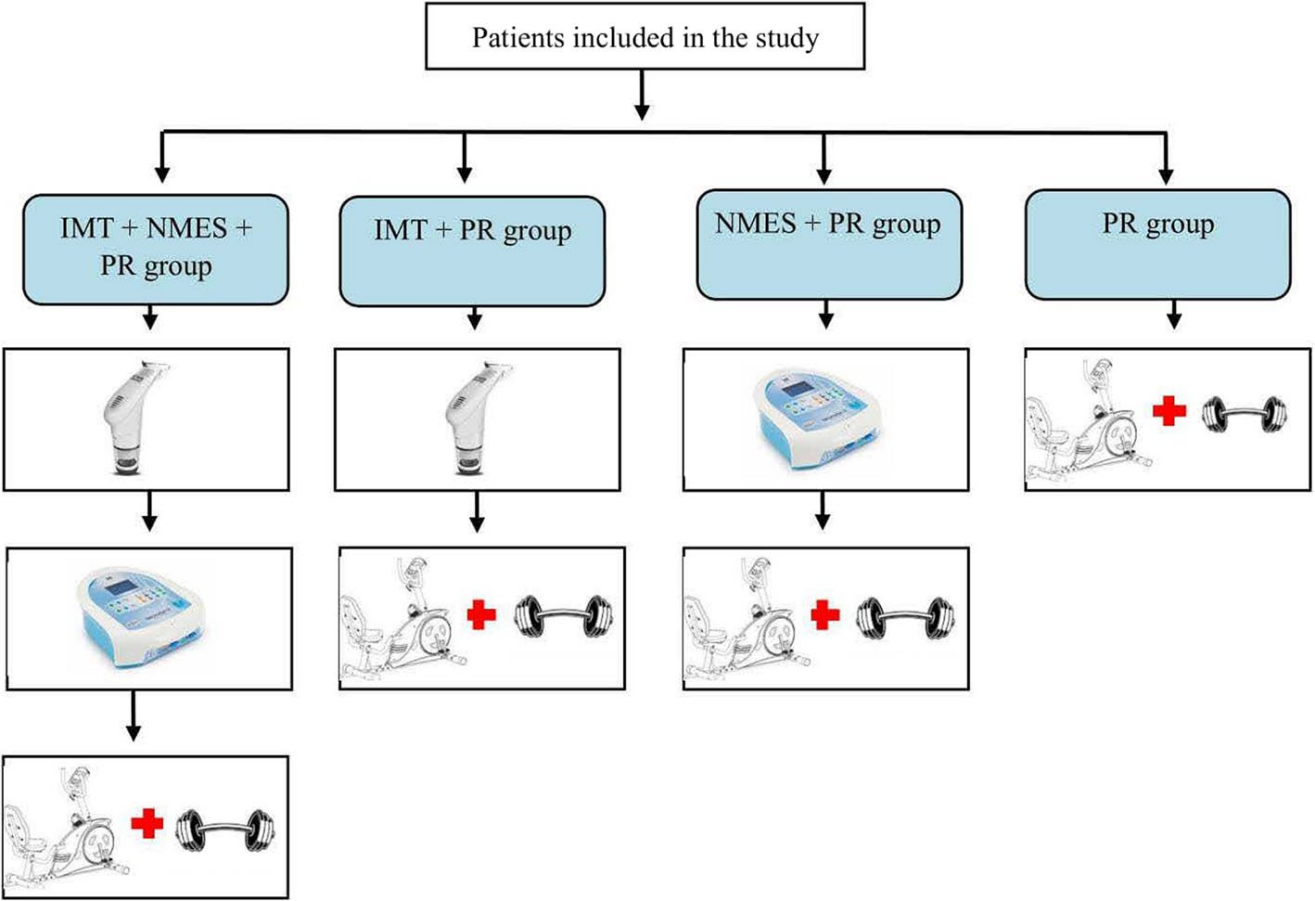
Sequence and type of therapeutic modality proposed in each study group. IMT: Inspiratory muscle training; NMES: Neuromuscular electrical stimulation; PR: Pulmonary rehabilitation

After baseline evaluations, all patients will receive the proposed therapeutic modality according to the group to which they will be randomly assigned (Fig. 2). All interventions will occur under the direct supervision of a physical therapist with more than 5 years of clinical experience, aiming to improve adherence to intervention protocols (blinded to outcome assessment), for 8 weeks, twice per week, for a total of 16 sessions. Vital signs will be continuously monitored, and possible adverse events will be recorded.

### Intervention description {11a}

#### Inspiratory muscle training

Patients randomized to the (IMT + NMES + PR) and (IMT + PR) groups will undergo IMT using the POWERbreathe Medic Plus (POWERbreathe Medic Plus®, SP, BR). The initial training load will be at 30% of the MIP (obtained by manovacuometry) during the first 2 weeks to allow for an adjustment period. Load increases will occur as follows: 35% of MIP in week 3, 40% of MIP in week 4, 45% of MIP in week 5, 50% of MIP at week 6, 55% of MIP in week 7, and 60% of MIP in weeks 8. The IMT will consist of five sets of 10 repetitions each, with a 1-min interval between each set, twice a week for 8 weeks. During training, patients will be instructed to maintain diaphragmatic breathing. In order to get feedback regarding perceived inspiratory effort, the modified Borg CR10 scale (4–6 out of 10) will be considered.

#### Neuromuscular electrical stimulation (NMES)

The (IMT + NMES + PR) and (NMES + PR) groups will receive the NMES for 16 sessions (2 days per week for 8 weeks). NMES will be applied bilaterally using an electrical stimulator (Neurodyn II, model N53, IBRAMED, SP, BR) with one proximal electrode (7.5 × 13 cm) at the motor point of the quadriceps muscle and another distal electrode above the upper pole of the patella [14]. The patient will be placed in the dorsal decubitus position, lower limbs on a foam wedge, and knees flexed at 60° [14]. The NMES protocol will consist of the application of symmetrical biphasic rectangular pulses, 50 Hz frequency, 400 ms pulse width, stimulation (ON) time of 5 s, and relaxation (OFF) time of 15 s (first month), and 10 s ON and 30 s OFF (second month). The maximum intensity tolerated progressively will be used, which should culminate in visible and comfortable muscle contraction [14, 15].

#### Pulmonary rehabilitation program

All patients will undergo the PR program for approximately 60 min per session. Firstly, aerobic training will be performed on a cycle ergometer (Kikos kr5.6 bivolt, SP, BR) for 30 min; the initial training intensity target will be 60% of the maximum work rate [16]. The increase in exercise intensity will be based on the rating of perceived exertion (4–6 on the modified Borg scale [8, 9]). Furthermore, the heart rate target will be kept between 50 and 80% of resting HR [8]. Three sets of eight repetitions will be performed at intensities initially defined as 50% of the one-repetition maximum (1RM) with weekly increases until it reaches 80% of the 1RM at the end of 8 weeks [8, 9].

### Criteria for discontinuing or modifying allocated interventions {11b}

Patient withdrawal from the study will only occur if consent is obtained. The presence of clinical instability is the criterion for discontinuing the interventions. However, patient follow-up will proceed normally; that is, the patient will not be excluded from the analyses (reassessments).

### Strategies to improve adherence to interventions {11c}

Patients will individually receive a report at the end of the study, with the performance obtained in the evaluations at pre- and post-intervention. Moreover, every session will be overseen by a researcher with experience in exercise rehabilitation to ensure that the patients perform the exercises properly.

### Relevant concomitant care permitted or prohibited during the trial {11d}

Participants are allowed to maintain their pharmacological treatment throughout the study. Participants will be excluded in case of any modification of the dose or type of drug during the trial. Furthermore, patients will be instructed not to perform any other form of exercise during the intervention.

### Provisions for post-trial care {30}

There was no anticipated harm or compensation for participating in the trial. At the end of the study, participants interested in continuing PR will be referred to the university hospital for outpatient care.

### Outcomes {12}

The results will be evaluated at two time points: (1) baseline (before starting the intervention program) and (2) after 8 weeks of intervention. Trained assessors will conduct all assessments and reassessments. The evaluators will be physical therapists with at least 1 year of training experience in the evaluation instruments used in this study.

#### Primary outcome

##### Static postural balance

The primary outcome will be measured using the AMTI portable force platform model OR6-6-2000 (Advanced Mechanical Technologies, Inc.) with an acquisition frequency of 100 Hz [17]. Standardized verbal instructions will be used to guide proper patient positioning. Three attempts lasting 30 seconds each, with 1 minute rest in between, will be performed (eyes open and then closed) and the average will be considered for analysis [18].

Body sway will be evaluated from the center of pressure (COP) and the variables will comprise COP anteroposterior displacement amplitude (COPap), COP mid-lateral displacement amplitude (COPml), COP displacement velocity (COPvel) and 95% ellipse area (AE). Then, the raw data obtained will be filtered using a fourth-order zero-lag Butterworth with 10 Hz cut-off frequency, and processed using custom Matlab routines (R2020a, The Mathworks, Inc., Massachusetts, USA).

#### Secondary outcomes

The secondary outcomes measured are static and dynamic postural balance, fear of falling, muscle strength and endurance (peripheral and respiratory), functional capacity, HRQoL, muscle architecture (quadriceps and diaphragm) and laboratory biomarkers.

##### Static postural balance

To assess vestibular, proprioceptive, and visual functions, dynamic flow-laser posturography will be used. Each evaluation will be performed three times for 20 seconds and considering for analysis the mean of the values obtained [19].

##### Dynamic postural balance

Dynamic postural balance will be assessed using the Timed Up and Go (TUG). Three tests will be performed and the best result one will be considered [20].

##### Static and dynamic postural balance

The Berg Balance Scale and the Balance Evaluation Systems Test (BESTest) will be also used to assess the static and dynamic postural balance [21, 22].

##### Assessment of fear of falling

The fear of falling will be assessed using the Falls Efficacy Scale-International-Brazil (FES-I-Brazil) [23] and the Activities-specific Balance Confidence (ABC) scale [24].

##### Respiratory muscle strength

The evaluation of respiratory muscle strength will be performed by means of measurements of maximal inspiratory pressure (MIP) and maximal expiratory pressure (MEP) with an MVD 300 digital manometer (MDI^®^, RS, BR). The highest pressure of MIP and MEP will be considered [25].

##### Inspiratory muscle endurance

The assessment of inspiratory muscle endurance will be performed by using POWERbreathe^®^ Medic Plus (POWERbreathe Medic Plus®, SP, BR), coupled to an analogical pressure transducer (WIKA, Alexander Wiegand SE & Co., Klingenberg, Germany). The evaluation will be composed of the incremental and constant tests [25].

##### Peripheral muscle strength

The assessment of upper limb muscle strength will be performed by a hydraulic dynamometer (Saehan Corporation SH5001, Korea) and the highest value obtained in each limb considered for analysis [26]. Lower limb muscle strength will be evaluated using a MicroFET® (Hoggan Health Industries, West Jordan, UT, USA) handheld dynamometer. Five measurements will be taken for each limb, of which the highest and lowest values will be discarded and the average between the three remaining values calculated [26].

##### Peripheral muscle endurance

The peripheral muscle endurance will be evaluated through the 30-s sit-to-stand test (30-STST) [27, 28]. The number of ≥ 2 repetitions in the test will be considered for analysis.

##### Functional capacity

Functional capacity will be evaluated by the six-minute walk test (6MWT) and the longest walk distance will be considered for analysis [29].

##### Health-related quality of life (HRQoL)

The HRQoL will be assessed using the Saint George's Respiratory Questionnaire [30].

##### Diaphragm and peripheral skeletal muscle architecture

Diaphragm and peripheral skeletal muscle architecture (cross-sectional area, muscle layer thickness and echogenicity) will be assessed with high-resolution US (Mindray Ultrasound, portable DP-2200, China), in B mode, with an echocardiologic transducer (65C15EA 5.0-9.0 MHz, 4W). The image analysis will be performed in ImageJ^®^ software (NIH, Bethesda, MD, USA) [31, 32].

##### Inflammatory biomarker

The hsCRP will be used as an inflammatory biomarker and calculated using standard method on a Mindray automated system (BS 380®, Shenzhen, China).

##### Endothelial function

The nitrate and nitrite levels will be used to assess endothelial function, on the Mindray automated system (BS 380®, Shenzhen, China), according to the modified Griess method by Tatsch et al. (2011) [33].

##### Oxidative stress

The oxidant profile will be analyzed by the Thiobarbituric Acid-Reactive Substances assay, advanced oxidation protein products, protein carbonylation test and the total oxidant status. The antioxidant profile will be measured by means of the total antioxidant capacity and the ferric reducing ability of plasma. The sample treatment will be based on established methods.

##### Muscular damage

Muscle damage will be evaluated by the Total creatine phosphokinase, lactate dehydrogenase and lactate that will be calculated using standard method on a Mindray automated system (BS 380®, Shenzhen, China).

##### DNA damage

The DNA damage will be measured by the comet assay and micronuclei analysis. We will select and analysis images of 100 cells from each sample according to tail length from 0 (no migration) to 4 (maximal migration), resulting in single DNA damage score (DNA damage index) [34]. Two different evaluators will analyze the slides under blind conditions [35]. Micronuclei analysis will be performed in peripheral blood lymphocytes as previously described [36].

### Participant timeline {13}

The participant timeline is shown in Table 1.

**Table 1:** Participant timeline

	**STUDY PERIOD**			
	**Enrolment**	**Allocation**	**Post-allocation**	**Close-out**
TIMEPOINT**	***-t***_***1***_	**0**	***t***_***1***_	***t***_***x***_
**ENROLMENT:**				
**Eligibility screen**	X			
**Informed consent**	X			
**Allocation**		X		
**INTERVENTIONS:**				
***IMT + NMES + PR group***			X	
***IMT + PR group***			X	
***NMES + PR group***			X	
***PR group***			X	
**ASSESSMENTS:**				
**Static and dynamic postural balance**	X			X
**Assessment of Fear of falling**	X			X
**Respiratory muscle strength**	X			X
**Inspiratory Muscle Endurance**	X			X
**Peripheral muscle strength**	X			X
**Peripheral muscle endurance**	X			X
**Functional capacity**	X			X
**Health-related quality of life**	X			X
**Laboratorial biomarkers**	X			X

### Sample size {14}

Based on the data from a previous study, performed by Mekki et al. [13] (*n*=45 participants, conducted in Tunisia), it will be necessary in a minimum sample size of 10 individuals per group and a total of 40 patients, considering the variable of mean lateral displacement of the pressure center mid-lateral displacement amplitude (COPml) in the follow-up of the intervention group, which performed NMES associated with PR program (±12.1 mm) and the control group that only performed standard PR (±7.2 mm). We also considered *p* value <0.05, power of 80% and loss of 20%.

### Recruitment {15}

Patients will be recruited from the waiting list for the PR program at the outpatient pulmonology clinic of the University Hospital of Santa Maria, Rio Grande do Sul, Brazil.

## Assignment of interventions: allocation

### Sequence generation {16a}

A computer-generated list of random numbers will be used, and a randomization sequence will be created using the Random Number Generator Pro v2.00 software (Segobit, Issaquah, WA, USA).

### Concealment mechanism {16b}

A single investigator blinded to patient identity (I.M.A.) will randomly divide patients into the four groups (1:1:1:1): the multimodal exercise program (IMT + NMES + PR group), (IMT + PR group), (NMES + PR group), or standard PR group.

### Implementation {16c}

All eligible patients who provide consent for participation and fulfill the inclusion criteria will undergo an initial study evaluation. Immediately after the initial assessments, the project manager (IMA) will complete the randomization and reveal the group allocation to the patient and physiotherapist responsible for performing the intervention protocols. Randomization will be conducted without any influence of the principal investigators, outcome assessors, or physiotherapists. Assessments will be conducted by independent assessors, blinded to group allocation. All data analyses will be blinded.

## Assignment of interventions: blinding

### Who will be blinded {17a}

This will be a single-blind study, in which the outcome assessors will have no information regarding the study group.

### Procedure for unblinding if needed {17b}

The design is open-label with only outcome assessors being blinded so unblinding will not occur.

## Data collection and management

### Plans for assessment and collection of outcomes {18a}

As previously described (item outcomes - 12), all assessments will be performed using instruments previously validated in the literature. At least three measurements will be performed for the performance tests. The evaluators will be physical therapists with at least 1 year of training experience in the evaluation instruments used in this study.

### Plans to promote participant retention and complete follow-up {18b}

The participants who enroll in the study in each session will be asked to provide feedback regarding their health status. Participants will receive a schedule with the dates and times for the assessment and training, in addition to a text message to remind them of the training schedule. An intention-to-treat analysis will be applied to include all randomized participants.

### Data management {19}

After inclusion, a unique identification code will be assigned to each participant. An Excel file including the individual codes and the corresponding participants will be stored by a leading researcher (IMA). All data collected/analyzed will be entered into an anonymized/coded Excel database. Consents and printed documents will be stored for 5 years after the end of the study and will be discarded thereafter.

### Confidentiality {27}

All laboratory specimens and data collection forms will be identified using coded ID numbers to maintain the participants’ confidentiality. None of the personal information of potential or enrolled participants will be shared or released.

### Plans for collection, laboratory evaluation and storage of biological specimens for genetic or molecular analysis in this trial/future use {33}

Blood samples will be collected after an 8-h overnight fast. Serum will be used for the analysis of high-sensitivity C-reactive protein (hsCRP), total antioxidant capacity, total oxidant status, nitrate and nitrite, creatine phosphokinase, and lactate dehydrogenase levels. Plasma will be used to assess the advanced oxidation protein products, the ferric reducing ability of plasma, Thiobarbituric Acid-Reactive Substances assay, protein carbonylation, DNA damage, and musculature. Biological samples will be stored at the Research Laboratories of Clinical Biochemistry and Biogenomics at “X”.

## Statistical methods

### Statistical methods for primary and secondary outcomes {20a}

The data will be analyzed using GraphPad Prism 5 (GraphPad Software Inc., San Diego, CA, USA). The normality of variables will be assessed by the Shapiro-Wilk test. The continuous variables with normal distribution and those with non-normal distribution will be presented as mean ± standard deviation (SD), 95% confidence interval (95% CI), and median (interquartile range), respectively, while categorical variables will be presented as absolute frequencies and percentages. Student’s *t* test for paired samples (parametric data) or Wilcoxon test (non-parametric data) will be used to compare the results before and after the intragroup intervention according to data distribution, and the effect size will be calculated using Cohen’s d. Comparison between groups will be performed using two-way analysis of variance (ANOVA) with repeated measures, followed by Bonferroni’s post hoc. Effect size was calculated using Cohen’s d. The significance level will be set at 5% (*p* <0.05).

### Interim analyses {21b}

No anticipated problems that are detrimental to the participant were considered for interruption of this study.

### Methods for additional analyses (e.g. subgroup analyses) {20b}

We will use analysis of covariance (ANCOVA), as a supportive analysis, to compare differences between groups after the intervention, adjusting for values of the respective outcomes at baseline.

### Methods in analysis to handle protocol non-adherence and any statistical methods to handle missing data {20c}

Intention-to-treat analysis will be applied to include all randomized participants. Thus, the participants will be analyzed in the groups to which they will be allocated, even if they do not complete the intervention protocol (reassessments are intended for all patients). If this is not possible, the missing data will be handled using a multiple imputation method.

### Plans to give access to the full protocol, participant-level data, and statistical code {31c}

The datasets analyzed during the current study and statistical code are available from the corresponding author upon reasonable request, as is the full protocol.

## Oversight and monitoring

### Composition of the coordinating center and trial steering committee {5d}

The center is coordinated by IMA and the principal investigator, TDS. The trial will be directed by the investigators, TDS and AFS. No additional steering committees were considered for this study. All researchers will attend weekly meetings to discuss research progress and possible unforeseen events. There is no stakeholder and public Involvement Group (SPIG).

### Composition of the data monitoring committee, its role and reporting structure {21a}

Data monitoring committee is not considered as this is a low-risk intervention.

### Adverse event reporting and harms {22}

Adverse events (AEs) are not anticipated. The potential minor AEs that may be anticipated are fatigue, tachycardia, desaturation, dizziness, or blurred vision during the exercise that will be minimized by monitorization before and after the assessment and intervention. If the symptoms are persistent, the physiotherapist will stop exercise immediately and will conduct the first aid. The details of AEs will be reported in the case report form (CRF). All protocol violations and all AEs could be registered and reported in the final paper and serious AEs will be reported within 24 h to the Ethics Committee of the Federal University of Santa Maria.

### Frequency and plans for auditing trial conduct {23}

The trial will be continuously monitored by the principal investigators and discussed weekly in meetings with all researchers involved. As this study was unicentric and small, and the interventions were considered to be of low-risk, supervision by an independent data monitoring committee was not required. No additional audits were conducted in this study. However, the researchers were committed to reporting any adverse events that occurred during the study to the Ethics Committee of the Federal University of Santa Maria.

### Plans for communicating important protocol amendments to relevant parties (e.g. trial participants, ethical committees) {25}

Any changes to the study protocol will be submitted to the local Research Ethics Committee and modifications will be updated at ClinicalTrials.gov.

### Dissemination plans {31a}

The results of this study will be communicated to health authorities, health professionals, and the general public, at events and through publications in scientific journals as soon as the results are available. Additionally, each participant will receive a full report on the results of their assessments.

## Discussion

To our knowledge, this is the first study to investigate the effects of a short-term multimodal exercise program on postural balance and other outcomes with clinical significance in patients with COPD. Previous studies have demonstrated that deficits in postural balance are increasingly recognized as important secondary impairments in patients with COPD [6, 7, 10]. In addition, many risk factors that lead to impaired postural balance are common in elderly patients and systemic manifestations of COPD. Therefore, we suggest that most individuals with COPD are affected twofold [37]. However, the description of PR components in the ATS/ERS Pulmonary Rehabilitation Statement does not address the use of resources or strategies focused on improving postural balance [8, 9].

In this context, it is necessary to expand the focus of PR to include assessments and therapeutic strategies to improve balance, as proposed in this study. Considering that COPD is a complex inflammatory disease involving several types of inflammatory cells and multiple inflammatory mediators [38], novel PR models should provide a comprehensive understanding that incorporates the physiological and functional implications of different intervention strategies in a clinical setting. Nevertheless, the current evidence on the effects of multimodal exercise program on postural balance in patients with COPD is a significant gap in the literature.

Based on the recent recommendations from the official ATS workshop report international guidelines, a new approach to the future of PR is required [38]. Consistent with these recommendations and to ensure the principle of personalized rehabilitation, a combination of interventions that include the use of IMT and NMES may be particularly relevant. Janssens et al. (2013) were pioneers in encouraging further studies to determine the effects of IMT on postural balance in patients with COPD [39]. Recently, a randomized controlled trial demonstrated that IMT combined with endurance training enhanced inspiratory muscle function and balance in these patients [12]. Additionally, NMES was able to reduce DNA damage in patients with chronic kidney failure [14] and added to PR improve static postural balance in patients with COPD [13]. In this sense, the results of this study will reveal relevant clinical information regarding the adoption of new PR models through a multimodal exercise program. These results can also be applied to clinical practice in the field of physical therapy.

Our study has both strengths and limitations. Strengths include the adoption of a multimodal exercise program because this strategy may promote additional benefits on clinically relevant outcomes; use of the protocol for short-term intervention; inclusion of laboratory biomarkers for inflammatory profile, endothelial function, oxidative stress, and DNA damage that had not been reported in previous studies comparing therapeutic strategies in COPD patients [40]. Moreover, we will implement a combination of strategy modalities considered to be low-cost and feasible. One limitation is that the study will be performed at a single center, and it is not possible to include multiple centers because of the type of intervention and inability to blind the therapist to the treatment allocation.

The results from will reveal relevant clinical information about the adoption of new PR models through multimodal exercise program. Thus allowing to expand the focus of PR to include the assessment and balance deficits training in COPD. Physical therapists will be able to transfer this knowledge in clinical practice to improve the treatment of these patients.

## Trial status

Completed

ClinicalTrials.gov NCT04387318. The protocol version is number 3.0, dated May, 28, 2023.

The first patient was recruited on August 01, 2021; the recruitment will be completed on December 20, 2022.

## Data Availability

The datasets generated during and/or analyzed during the current study will be made available with other researchers on duly justified request. The authors TDS, DMC, AFS and IMA will have access to the final test data set.

## Abbreviations

ABC: Activities-specific Balance Confidence Scale
AE: 95% ellipse area
ANCOVA: Covariance
ANOVA: Two-way analysis of variance
ATS: American Thoracic Society
BESTest: Balance Evaluation Systems Test
CRF: Case report form
CONSORT: Consolidated Standards of Reporting Trials
COP: Center of pressure
COPap: COP anteroposterior displacement amplitude
COPD: Chronic obstructive pulmonary disease
COPml: COP mid-lateral displacement amplitude
COPvel: COP displacement velocity
ERS: European Respiratory Society
FES-I-Brazil: Falls Efficacy Scale-International-Brazil
HRQoL: Health-related quality of life
hsCRP: High-sensitivity C-reactive protein
IMT: Inspiratory muscle training
MEP: Maximal expiratory pressure
MIP: Maximal inspiratory pressure
NMES: Neuromuscular electrical stimulation
95% CI: 95% confidence interval
PR: Pulmonary rehabilitation
6MWT: Six-minute walk test
SD: Standard deviation
30-STST: 30-s sit-to-stand test
TUG: Timed Up and Go
1RM: One-repetition maximum
